# Evaluation of Bioactive Compounds, Antioxidant Activity, and Anticancer Potential of Wild *Ganoderma lucidum* Extracts from High-Altitude Regions of Nepal

**DOI:** 10.3390/cimb47080624

**Published:** 2025-08-05

**Authors:** Ishor Thapa, Ashmita Pandey, Sunil Tiwari, Suvash Chandra Awal

**Affiliations:** 1 Department of Biotechnology, SANN International College, Purbanchal University, Biratnagar 56600, Nepal; ishor.thapa@hci.utah.edu (I.T.); pandey.ashmeeta@gmail.com (A.P.); suvash.awal@gmail.com (S.C.A.); 2 Department of Oncological Sciences, University of Utah School of Medicine, Salt Lake City, UT 84132, USA; 3 School of Life Sciences, Arizona State University, Tempe, AZ 85281, USA

**Keywords:** *Ganoderma lucidum*, solvent extraction, antioxidant activity, cytotoxicity, high-altitude fungi, DPPH, MTT assay, taxonomy, GC-MS

## Abstract

Wild *Ganoderma lucidum* from Nepal’s high-altitude regions was studied to identify key bioactive compounds and assess the influence of solvent type—water, ethanol, methanol, and acetone—on extraction efficiency and biological activity. Extracts were evaluated for antioxidant potential, cytotoxicity against HeLa cells, and phytochemical composition via gas chromatography–mass spectrometry (GC-MS). Solvent type significantly affected both yield and bioactivity. Acetone yielded the highest crude extract (5.01%), while ethanol extract exhibited the highest total phenolic (376.5 ± 9.3 mg PG/g) and flavonoid content (30.3 ± 0.5 mg QE/g). Methanol extract was richest in lycopene (0.07 ± 0.00 mg/g) and β-carotene (0.45 ± 0.02 mg/g). Ethanol extract demonstrated consistently strong DPPH, superoxide, hydroxyl, and nitric oxide radical scavenging activity, along with high reducing power. All extracts showed dose-dependent cytotoxicity against HeLa cells, with ethanol and water extracts showing the greatest inhibition (>65% at 1000 µg/mL). GC-MS profiling identified solvent-specific bioactive compounds including sterols, terpenoids, polyphenols, and fatty acids. Notably, pharmacologically relevant compounds such as hinokione, ferruginol, ergosterol, and geranylgeraniol were detected. These findings demonstrate the therapeutic potential of *G. lucidum*, underscore the importance of solvent selection, and suggest that high-altitude ecological conditions may influence its bioactive metabolite profile.

## 1. Introduction

*Ganoderma lucidum*, commonly known as “Lingzhi” in China, “Reishi” in Japan, and “Dadu chyau” in Nepal, is a polypore mushroom with a long and storied history of use in East Asian medicine for promoting health and longevity [1]. It is often referred to as the “King of Herbs” or the “Mushroom of Immortality”. Its medicinal use dates back over 2000 years, with its effects documented in ancient scripts like the *Shen Nong Ben Cao Jing* from China’s Eastern Han dynasty (25–220 AD) [2]. Traditionally, it has been used to treat a variety of ailments and is believed to enhance stamina, increase brain power, improve circulation, and strengthen the immune system [3].

Modern research has begun to validate these traditional claims, attributing the mushroom’s medicinal properties to its rich and varied chemical composition. Bioactive compounds such as polysaccharides, triterpenes, adenosine, organic germanium, phenolic compounds, flavonoids, and ergosterol contribute to its therapeutic effects, which include antioxidant, anticancer, anti-inflammatory, and antimicrobial activities [4,5,6,7,8]. For instance, triterpenic acids have demonstrated significant anticancer effects, while polysaccharides have shown anti-diabetic and antibiotic properties [9,10,11,12]. The antioxidant properties of *G. lucidum* are particularly noteworthy, as they neutralize reactive oxygen species (ROS) and reactive nitrogen species (RNS), which are implicated in the development of chronic diseases such as cancer, aging, diabetes, cardiovascular disorders, and neurodegeneration [4,13]. Studies have shown that polysaccharides in *G. lucidum* exhibit potent ROS-scavenging activity and can inhibit tumor growth through immunomodulation and the induction of apoptosis [6,9,10]. Furthermore, its anti-inflammatory effects have been observed in animal models, suggesting significant therapeutic potential [14].

Nepal, with its diverse geography ranging from the Terai plains to the high Himalayas, provides a unique environment for a wide variety of mushrooms [15,16,17,18,19]. While approximately 1300 mushroom species have been reported in Nepal, with 73 identified as having medicinal value, the therapeutic potential of most, including native *G. lucidum*, remains largely unexplored [20,21]. Research on Nepalese mushrooms, initiated by the Nepal Agricultural Research Council (NARC) in 1974, has historically focused more on cultivation and taxonomy rather than on the detailed analysis of their bioactive properties.

**Figure 1:**
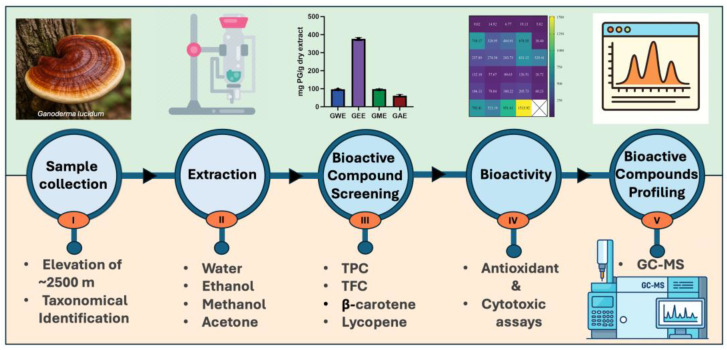
Overview of the study workflow. *Ganoderma lucidum* fruiting bodies were collected from a high-altitude site (~2500 m) and taxonomically identified (I). Crude extracts were prepared using four solvents: water, ethanol, methanol, and acetone (II). Bioactive compounds were screened by quantifying total phenolics (TPC), flavonoids (TFC), and carotenoids (III). Antioxidant and cytotoxic assays were conducted to evaluate bioactivity (IV). Chemical constituents of the extracts were further identified using gas chromatography–mass spectrometry (GC–MS) (V).

The unique environmental conditions of Nepal’s high-altitude regions—characterized by lower oxygen levels, intense UV radiation, and distinct soil compositions—may lead to the production of *G. lucidum* strains with enhanced or novel bioactive profiles [22,23]. Despite Nepal’s rich biodiversity, research on high-altitude *G. lucidum* is scarce, leaving a gap in our understanding of its full potential. This study, therefore, investigated the bioactive constituents, antioxidant activities, and cytotoxic effects of wild *G. lucidum* collected from the high altitudes of Nepal (Figure 1), utilizing various solvent extracts to comprehensively characterize its mycochemical composition and therapeutic promise.

## 2. Materials and Methods

### 2.1. Materials

2,2-Diphenyl-1-picrylhydrazyl (DPPH, ≥95%), pyrogallol (≥98%), Folin–Ciocalteu reagent (2M), sodium carbonate (Na_2_CO_3_, ≥99.5%), aluminum chloride (≥99%), potassium acetate (≥99%), quercetin (≥95%), ferrous sulfate (FeSO_4_, ≥99%), salicylic acid (≥99%), potassium ferricyanide (K_3_Fe(CN)_6_, ≥99%), and ferric chloride (FeCl_3_, ≥98%) were obtained from Sigma-Aldrich Co. (Burlington, MA, USA). Ascorbic acid (≥99%), trichloroacetic acid (99%), NP-40, and isopropanol (≥99%) were purchased from Thermo Scientific Chemicals (Waltham, MA, USA). The Griess reagent (≥98%), hydrochloric acid (HCl, >36.5%), hydrogen peroxide (H_2_O_2_), methanol, ethanol, acetone, and hexane were sourced from Fisher Scientific (Waltham, MA, USA) and were of analytical grade. Sodium nitroprusside (≥99%) and MTT [3-(4,5-dimethylthiazol-2-yl)-2,5-diphenyl tetrazolium bromide, ≥98%] were obtained from Merck Millipore (Darmstadt, Germany).

For solvent removal, a Buchi R-215 rotary vacuum evaporator equipped with a V-850 vacuum controller (BUCHI Corporation, New Castle, DE, USA) and a Multi-Vapor P-6 heating bath (BUCHI Corporation, New Castle, DE, USA) was used for simultaneous solvent evaporation under reduced pressure. Absorbance measurements for antioxidant assays were performed using a Labindia Analytical Double Beam UV 3200 Spectrophotometer (Labindia Analytical, Maharashtra, India). Cell culture reagents, including Dulbecco’s Modified Eagle Medium (DMEM), fetal bovine serum (FBS), and penicillin–streptomycin, were obtained from Gibco (Thermo Fisher Scientific, Waltham, MA, USA).

### 2.2. Collection and Identification

**Figure 2:**
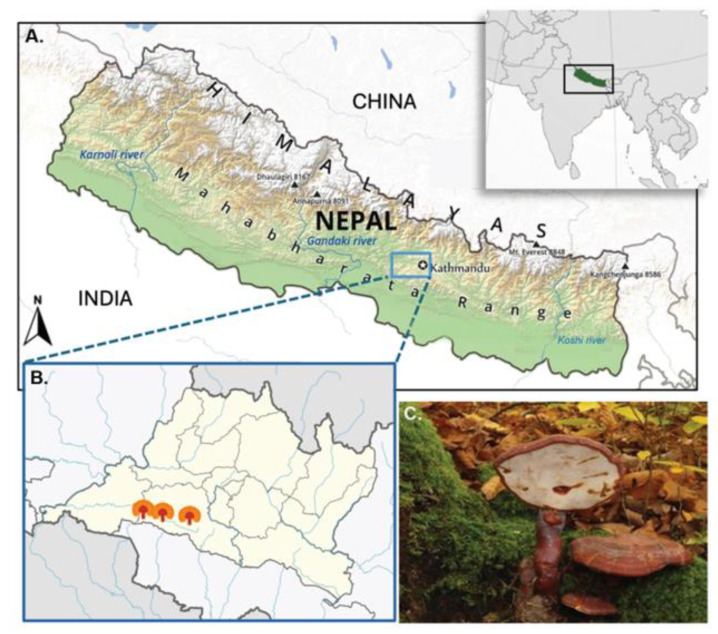
Map of Nepal in a global context (**A**), *G. lucidum* collection site (**B**), and its fruiting body growing on *Quercus lanata* (**C**).

Fresh fruiting bodies of wild *G. lucidum* were collected from a dead trunk of *Quercus lanata* on Chandragiri Hill, Nepal (elevation 7482 ft; latitude 27.67402; longitude 85.19874) (Figure 2). The specimens were identified based on detailed macro- and micro-morphological characteristics (Table 1).

### 2.3. Sample Preparation and Extraction

The samples were cleaned and oven-dried gradually from 45 °C to 60 °C over 3 days until a constant weight was attained. The dried mushrooms were milled into a fine powder and stored in airtight containers for future use. For extraction, 10 g of dried *G. lucidum* powder, pooled from multiple wild-collected fruiting bodies, was used. Solvent extraction was performed for 10 h in a Soxhlet apparatus with 250 mL each of water (GWE, 100 °C), 70% ethanol (GEE, 60 °C), 80% methanol (GME, 70 °C), and 50% acetone (GAE, 50 °C). Extracts were concentrated under vacuum in a rotary evaporator (50 °C) as shown in the Supplementary Figure S1 and stored in dark vials at 4 °C. The % yield for each solvent was calculated using the following formula:

$$\%\;Yield=\frac{weight\;of\;the\;G.\;lucidum\;powder\;before\;extraction\;(gm)}{weight\;of\;the\;obtained\;extract\;(gm)}\times 100$$

### 2.4. Estimation of Total Phenolic, Flavonoid, β-Carotene, and Lycopene

#### 2.4.1. Total Phenolic Content

Total phenolic content was determined using the Folin–Ciocalteu method with pyrogallol as the standard [24]. A calibration curve was established by measuring the absorbance of pyrogallol standards (10–100 µg/mL) at 760 nm (Supplementary Figure S2). Sample solutions (1 mL) were mixed with 5 mL of 10% Folin–Ciocalteu reagent for 5 min at room temperature. Subsequently, 4 mL of 15% Na_2_CO_3_ solution was added, and samples were vigorously mixed and incubated in the dark for 2 h at room temperature. Absorbance was read at 760 nm, and results were expressed as pyrogallol equivalents (mg/g dry extract).

#### 2.4.2. Total Flavonoid Content

Total flavonoid content was determined using the aluminum chloride colorimetric method, as adapted from Shraim et al., 2021 [25]. A quercetin standard curve was prepared by diluting a 10 mg quercetin stock in 50% methanol to concentrations ranging from 10 to 100 µg/mL, with absorbance measured at 415 nm (Supplementary Figure S3). Sample aliquots (1 mL) were mixed with 0.5 mL of 1.2% aluminum chloride, 0.5 mL of 120 mM potassium acetate, and 1 mL of distilled water. After 30 min of incubation at room temperature, absorbance was read at 415 nm. Results were expressed as mg quercetin equivalents per g of dry extract (mg QE/g dry weight).

#### 2.4.3. Estimation of β-Carotene and Lycopene Content

β-carotene and lycopene were determined as per Prakash et al., 2016 [26]. Dried extracts (100 mg) were extracted with 10 mL of acetone-hexane (4:6) for 1 min, then filtered. Filtrate absorbance was measured at 453, 505, 645, and 663 nm. Carotenoid concentrations were calculated using the following equations:

Lycopene = (−0.0458 × *A*_663_) + (0.372 × *A*_505_) + (0.0806 × *A*_453_) β-carotene = (0.216 × *A*_663_) − (0.304 × *A*_505_) + (0.452 × *A*_453_)

Results are presented as mg carotenoid per g of dry extract (mg carotenoid/g dry extract).

### 2.5. Determination of In Vitro Antioxidant Activities

#### 2.5.1. DPPH (2,2-Diphenyl-1-Picryl-Hydrazyl) Assay

Different concentrations of extracts (20–100 µg/mL) were prepared. To 1 mL of each extract concentration, 2 mL of ice-cold 0.1 mM DPPH solution was added. The mixtures were incubated in the dark at room temperature for 30 min. Absorbance was then measured at 517 nm against a methanol blank [27]. A 3 mL DPPH solution was used as the control. Percentage inhibition was calculated using the formula:

% inhibition = [(A_0_ − A_1_)/(A_0_)] × 100

where A_0_ is the absorbance of the control, and A_1_ is the absorbance of the sample.

#### 2.5.2. Superoxide Radical Scavenging Assay

Superoxide radical scavenging activity was assessed by a modified pyrogallol auto-oxidation method [28]. The reaction mixture contained 4.5 mL of 50 mM Tris-HCl buffer (pH 8.2), 0.4 mL of 25 mM pyrogallol, and 1 mL of sample (0.1–0.5 mg/mL). After 5 min incubation at 25 °C, the reaction was terminated by adding 1 mL of 8 mM HCl. Absorbance was measured at 420 nm. Ascorbic acid served as the positive control. Superoxide radical scavenging activity was calculated using the formula:

% inhibition = [(A_0_ − A_1_)/(A_0_)] × 100

where A_0_ is the absorbance of the control, and A_1_ is the absorbance of the sample.

#### 2.5.3. Hydroxyl Radical Scavenging Assay

Hydroxyl radical scavenging activity was assessed by measuring the inhibition of salicylic acid hydroxylation [28]. The 7 mL reaction mixture contained 1 mL of sample/standard (100–500 µg/mL), 2 mL of 6 mM FeSO_4_, 2 mL of 6 mM H_2_O_2_, and 2 mL of 6 mM salicylic acid. After incubation at 37 °C for 1 h, absorbance was measured at 510 nm due to the color change of salicylic acid. Scavenging activity was calculated as follows:

% inhibition = [(A_0_ − A_1_)/(A_0_)] × 100

where A_0_ is the absorbance of the control, and A_1_ is the absorbance of the sample.

#### 2.5.4. Nitric Oxide Radical Scavenging Assay

Nitric oxide radical scavenging activity was determined with slight modification from Alam et al., 2013 [29]. Samples or ascorbic acid (20–100 µg/mL, 1 mL) were mixed with 2 mL of 10 mM sodium nitroprusside in phosphate buffer and incubated at 25 °C for 2.5 h. To 3 mL of the incubated solution, 3 mL of Griess reagent (1% sulfanilamide, 0.1% naphthylethylenediamine dihydrochloride in 2% H_3_PO_3_) was added. Absorbance of the pink color was measured at 540 nm against a blank. Ascorbic acid served as a positive control. Percentage inhibition was calculated using the following equation:

% inhibition = [(A_0_ − A_1_)/(A_0_)] × 100

where A_0_ is the absorbance of the control, and A_1_ is the absorbance of the sample.

#### 2.5.5. Reducing Power Assay

The reducing power of samples was assessed via the ferric reducing antioxidant power (FRAP) assay [30]. One mL of sample or standard (20–100 µg/mL) was combined with 2.5 mL of 0.2 M phosphate buffer (pH 6.6) and 2.5 mL of 1% (*w*/*v*) K_3_Fe(CN)_6_. After 20 min incubation at 50 °C in a water bath, 2.5 mL of 10% (*w*/*v*) trichloroacetic acid was added to terminate the reaction. The mixture was centrifuged at 3000 rpm for 10 min. A 2.5 mL aliquot of the supernatant was then mixed with 2.5 mL distilled water and 0.5 mL of 0.1% (*w*/*v*) FeCl_3_. Absorbance was measured at 700 nm against a blank.

### 2.6. MTT Cell Viability Assay

HeLa cells were obtained from the American Type Culture Collection (ATCC, Manassas, VA, USA; Catalog no. CCL-2). Cells were maintained in Dulbecco’s Modified Eagle Medium (DMEM) supplemented with 10% fetal bovine serum and 1% penicillin–streptomycin at 37 °C in a humidified atmosphere containing 5% CO_2_. For the MTT assay, approximately 5 × 10^3^ cells were seeded per well in a 96-well plate. After 24 h, cells were treated with dimethyl sulfoxide or various extract concentrations for 48 h. Post-treatment, media were removed and replaced with 100 µL fresh medium containing the MTT (3-(4,5-dimethylthiazol-2-yl)-2,5-diphenyltetrazolium bromide) reagent (final concentration 0.4 mg/mL). Plates were incubated at 37 °C for 3 h, during which intracellular purple formazan was observed. Next, 100 µL of solubilization solution (4 mM HCl, 0.1% NP-40 in isopropanol) was added, and plates were kept in the dark for 15 min at room temperature. Absorbance was measured at 570 nm using a microplate reader [31]. Percentage inhibition was calculated as follows:

% inhibition = 100 − [(A_1_/A_0_) × 100]

where A_0_ is the absorbance of the control, and A_1_ is the absorbance of sample.

### 2.7. Gas Chromatography–Mass Spectrometry (GC-MS) Analysis

GC-MS analysis of *G. lucidum* extracts was conducted using a GCMS-QP2010 Ultra (Shimadzu, Kyoto, Japan) equipped with an Rtx-5 M5 capillary column (30 m × 0.25 mm, 0.25 µm film thickness; Restek, Bellefonte, PA, USA). The operating conditions, including solvent cut-off, temperature program, and MS scan parameters, were identical to those described by Tiwari et al., 2023 [32]. Detailed information on the GC-MS run-time procedures and analytical parameters are provided in the Supplementary Materials. Compounds were identified using NIST libraries (NIST 14, Gaithersburg, MD, USA).

### 2.8. Statistical Analysis

Statistical analysis was performed using GraphPad Prism 10 (GraphPad Software, San Diego, CA, USA). All experiments were conducted in three biological replicates, expressed as mean ± standard deviation (SD). One-way ANOVA with Tukey’s post hoc test was performed to test the statistical significance between control and experimental groups (*p* < 0.05). IC_50_ values were calculated using the built-in “Inhibitor vs. Response” nonlinear regression model in GraphPad Prism. Exact *p*-values for total phenolic and flavonoid content, β-carotene and lycopene content, antioxidant assays, and cytotoxicity assays are provided in Supplementary Figures S4–S11.

## 3. Results

### 3.1. Extraction Yield

Extraction efficiency is affected by the chemical nature of bioactive compounds, the extraction method used, sample particle size, the solvent used, as well as the presence of interfering substances [33,34]. The yield of extraction depends on the solvent with varying polarity, temperature, pH, extraction time, and composition of the sample [33,34]. Extraction efficiency (% yield) varied significantly (*p* < 0.0001) among solvents, with acetone yielding the highest crude extract (GAE; 5.01%), followed by ethanol (GEE; 3.43%), methanol (GME; 2.98%), and water (GWE; 2.29%) (Table 2).

### 3.2. Estimation of Total Phenolic and Flavonoid Content

**Figure 3:**
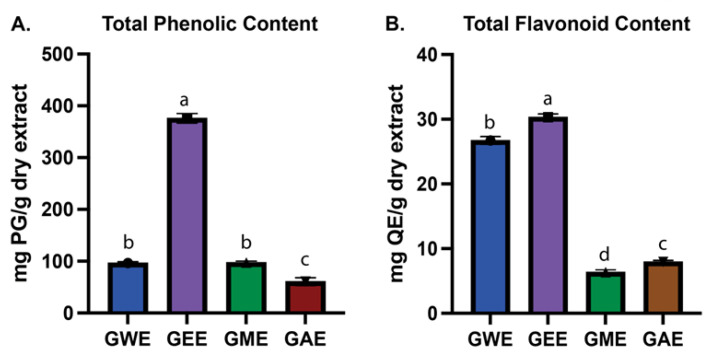
Total phenolic content (**A**) and total flavonoid content (**B**) expressed as pyrogallol and quercetin equivalents (mg/g dry extract), respectively. Data are mean ± SD from three biological replicates. Statistical analysis was performed using one-way ANOVA with Tukey’s multiple comparisons test (*p* < 0.05). Bars with the same letter (a–d) are not significantly different; different letters indicate significant differences between solvents.

Phenolic compounds are recognized as potent chain-breaking antioxidants due to the radical-scavenging capabilities of their hydroxyl groups [35]. The total phenolic content (TPC) exhibited significant variation among the tested solvents (Figure 3A). Ethanol extract (GEE) demonstrated the highest TPC (376.5 ± 9.3 mg PG/g), which was significantly greater (*p* < 0.0001) than that of methanol extract (GME; 97.3 ± 2.8 mg PG/g), water extract (GWE; 96.6 ± 2.6 mg PG/g), and acetone extract (GAE; 60.5 ± 7.4 mg PG/g). However, no significant difference was observed between GWE and GME (*p* = 0.9987), indicating comparable phenolic content in these two extracts (see Supplementary Figure S4).

Flavonoid content was quantified using the aluminum chloride colorimetric method and also showed significant variation among extracts (Figure 3B). GEE (30.3 ± 0.5 mg QE/g extract) and GWE (26.7 ± 0.6 mg QE/g extract) had the highest total flavonoid content, significantly (*p* < 0.0001) exceeding those of GME (6.3 ± 0.4 mg QE/g extract) and GAE (7.9 ± 0.2 mg QE/g extract). Notably, GEE contained approximately 4.7-fold higher total flavonoids compared to GME. All comparisons for TFC were statistically significant (*p* < 0.01), confirming solvent-dependent differences in flavonoid recovery (Supplementary Figure S4).

### 3.3. Estimation of β-Carotene and Lycopene

**Figure 4:**
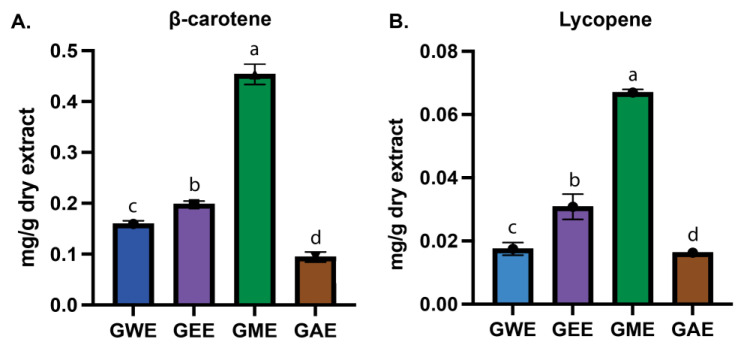
β-carotene (**A**) and lycopene (**B**) content as mg carotenoid per g of dry extract in various solvent extract. Data are mean ± SD from three biological replicates. Statistical analysis was performed using one-way ANOVA with Tukey’s multiple comparisons test (*p* < 0.05). Bars with the same letter (a–d) are not significantly different; different letters indicate significant differences between solvents.

The concentrations of lycopene and β-carotene in *G. lucidum* extracts were estimated spectrophotometrically. Carotenoid analysis demonstrated limited solvent efficacy and significant variation in extraction efficiency among solvents (Figure 4A,B). β-carotene content was highest in GME (0.45 ± 0.02 mg/g), followed by GEE (0.20 ± 0.01 mg/g), GWE (0.16 ± 0.00 mg/g), and GAE (0.09 ± 0.01 mg/g) (Figure 4A). Statistical analysis confirmed significant differences across most pairwise comparisons (*p* < 0.0001), with lesser degree between GWE and GEE (*p* = 0.0174) (see Supplementary Figure S4).

Similarly, lycopene content varied considerably among the extracts, ranging from 0.016 ± 0.000 to 0.067 ± 0.001 mg/g of dry extract (Figure 4B). The highest lycopene content was found in the methanolic extract (GME; 0.067 ± 0.001 mg/g), followed by the ethanolic extract (GEE; 0.031 ± 0.004 mg/g), water extract (GWE; 0.018 ± 0.002 mg/g), and acetone extract (GAE; 0.016 ± 0.000 mg/g). All pairwise comparisons showed statistically significant differences (*p* < 0.0001), except between GWE and GAE.

### 3.4. Comparative In Vitro Antioxidant Activities

Antioxidant activity cannot be definitively concluded from a single assay due to the diverse mechanisms involved and variations between in vitro test models. These diverse mechanisms include free radical scavenging, metal ion chelation, reducing power, single electron transfer, and others [29,36]. Therefore, this study employed multiple in vitro antioxidant assays (DPPH radical scavenging, superoxide radical scavenging, hydroxyl radical scavenging, nitric oxide radical scavenging, and reducing power) to comprehensively evaluate and compare the antioxidant potential of *G. lucidum* solvent extracts.

#### 3.4.1. DPPH Radical Scavenging Activity

**Figure 5:**
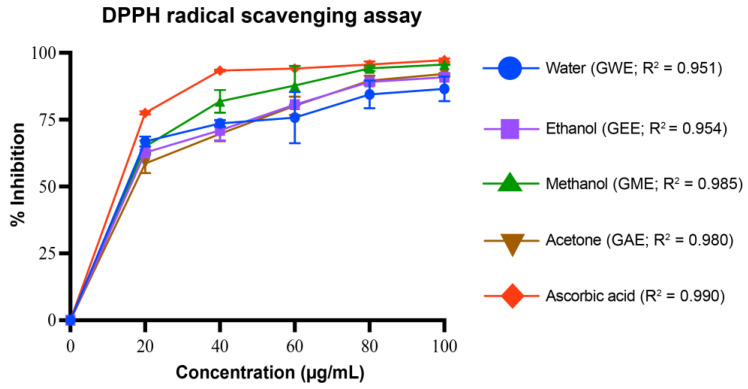
DPPH radical scavenging activity (%) of *G. lucidum* extracts prepared with four different solvents, compared to the standard antioxidant ascorbic acid. Dose–response regression lines with corresponding R^2^ values are shown. Data are mean ± SD from three biological replicates. Statistical analysis was conducted using one-way ANOVA with Tukey’s multiple comparisons test (*p* < 0.05). Exact *p*-values indicating significant difference between extracts at each concentration are provided in Supplementary Figure S5.

The DPPH radical scavenging activity of the extracts (at 100 µg/mL) ranged from 87 ± 5% to 96 ± 0.2% (Figure 5). Methanolic extract (GME) exhibited the highest activity (96 ± 0.2%), followed by acetone extract (GAE; 92 ± 0%), ethanolic extract (GEE; 92 ± 0%), and water extract (GWE; 87 ± 5%). Ascorbic acid, as a standard, showed 97 ± 1% inhibition. All extracts demonstrated strong radical scavenging activity in a dose-dependent manner, with scavenging over 80% of the DPPH radical even at 80 µg/mL. The regression analysis yielded strong R^2^ values for all extracts, ranging from 0.951 to 0.990, indicating high linearity in the dose–response relationships.

#### 3.4.2. Superoxide Radical Scavenging Activity

**Figure 6:**
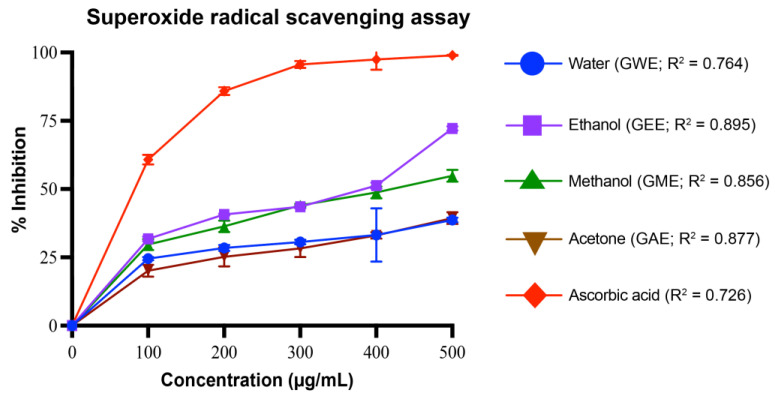
Superoxide radical scavenging activity (%) of *G. lucidum* extracts prepared with four different solvents, compared to the standard antioxidant ascorbic acid. Dose–response regression lines with corresponding R^2^ values are shown. Data are mean ± SD from three biological replicates. Statistical analysis was conducted using one-way ANOVA with Tukey’s multiple comparisons test (*p* < 0.05). Exact *p*-values indicating significant difference between extracts at each concentration are provided in Supplementary Figure S6.

Superoxide radical scavenging activity increased significantly with extract concentration (*p* < 0.05), demonstrating a dose-dependent response (Figure 6). At 500 µg/mL, GEE exhibited the highest scavenging activity (72 ± 1%), followed by GME (55 ± 2%), GAE (40 ± 2%), and GWE (39 ± 1%). Ascorbic acid showed 99 ± 0% scavenging. GEE was significantly more active than GWE, GME, and GAE (*p* < 0.0001), while GWE and GAE showed similar scavenging patterns, particularly at higher concentrations, and were significantly less active than GEE and GME (*p* < 0.0001). The scavenging efficiency followed the order: GEE > GME > GAE > GWE. Regression analysis of dose–response curves showed moderate to strong linearity, with R^2^ values ranging from 0.726 (ascorbic acid) to 0.895 (GEE). Among all extracts, GEE (R^2^ = 0.895) showed the best-fitting curve, followed by GAE (0.877), GME (0.856), and GWE (0.764).

#### 3.4.3. Hydroxyl Radical Scavenging Activity

**Figure 7:**
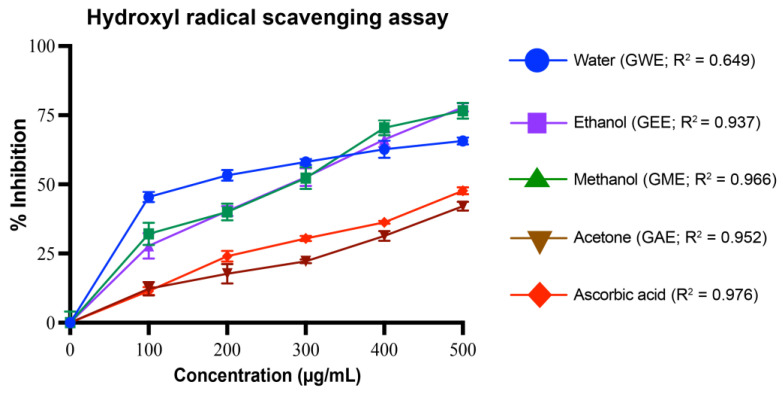
Hydroxyl radical scavenging activity (%) of *G. lucidum* extracts prepared with four different solvents, compared to the standard antioxidant ascorbic acid. Dose–response regression lines with corresponding R^2^ values are shown. Data are mean ± SD from three biological replicates. Statistical analysis was conducted using one-way ANOVA with Tukey’s multiple comparisons test (*p* < 0.05). Exact *p*-values indicating significant difference between extracts at each concentration are provided in Supplementary Figure S7.

All *G. lucidum* extracts exhibited significant, dose-dependent hydroxyl radical scavenging activity (*p* < 0.001) (Figure 7). At the tested concentrations, GME showed the highest activity (78 ± 1.5%), closely followed by GEE (77 ± 2.8%), and GWE (66 ± 1.3%), with GAE showing comparatively lower activity (42 ± 1.59%). The standard, ascorbic acid, achieved 48 ± 1.3% inhibition under same conditions, which was notably lower than that of GME, GEE, and GWE. Importantly, the IC_50_ values for GWE, GEE, and GME were lower than that of ascorbic acid, indicating their superior hydroxyl radical scavenging potential. Although GWE had a relatively low R^2^ value (0.0649), it displayed robust activity, particularly at lower concentrations, whereas GME, GEE, and GAE showed stronger linearity in their dose–response curves, with R^2^ values of 0.966, 0.937, and 0.952, respectively.

#### 3.4.4. Nitric Oxide Radical Scavenging Activity

**Figure 8:**
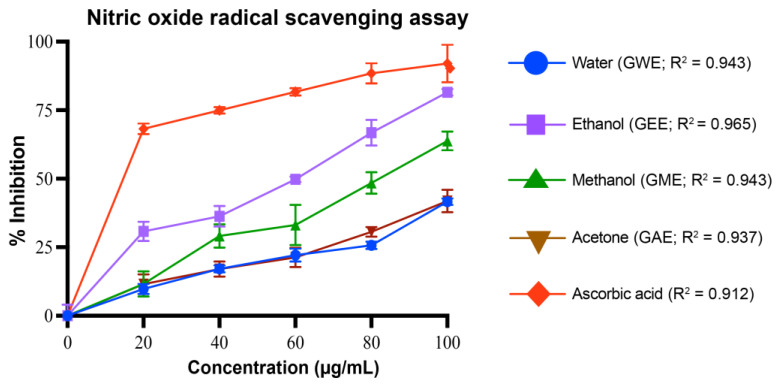
Nitric oxide radical scavenging activity (%) of *G. lucidum* extracts prepared with four different solvents, compared to the standard antioxidant ascorbic acid. Dose–response regression lines with corresponding R^2^ values are shown. Data are mean ± SD from three biological replicates. Statistical analysis was conducted using one-way ANOVA with Tukey’s multiple comparisons test (*p* < 0.05). Exact *p*-values indicating significant difference between extracts at each concentration are provided in Supplementary Figure S8.

The *G. lucidum* extracts demonstrated good inhibition of nitric oxide radicals in a concentration-dependent manner (Figure 8). Among them, GEE was significantly more active than GME, GAE, and GWE (*p* < 0.0001). At 100 µg/mL, GEE exhibited the highest scavenging potential (82 ± 1.2%), followed by GME (64 ± 3.4%), while GAE (42 ± 2.8%), and GWE (42 ± 2.9%) showed statistically similar lower activities (*p* > 0.999). Ascorbic acid showed 92 ± 6.9% inhibition. GEE and ascorbic acid showed no significant difference (*p* = 0.0523) at 100 µg/mL despite the numerical difference in inhibition. The order of activity was GEE > GME > GAE ≈ GWE. Regression analysis of the dose–response curve revealed strong linearity for all extracts. Among the extracts, GEE, GME, GWE, and GAE showed consistent trends, supporting the reliability of the observed dose-dependent activity.

#### 3.4.5. Reducing Power Assay

**Figure 9:**
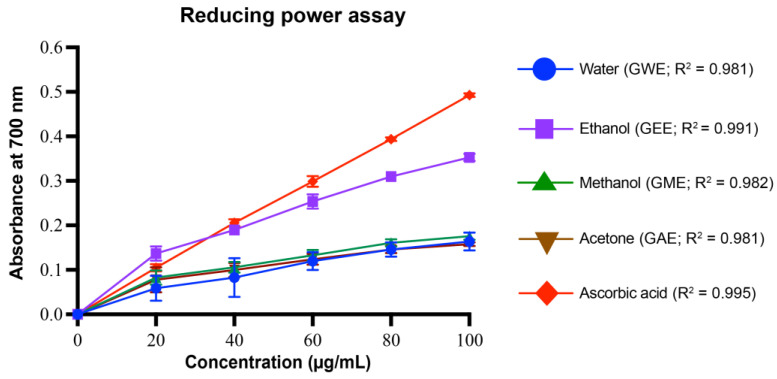
Reducing power assay of *G. lucidum* extracts prepared with four different solvents, compared to the standard antioxidant ascorbic acid. Dose–response regression lines with corresponding R^2^ values are shown. Data are mean ± SD from three biological replicates. Statistical analysis was conducted using one-way ANOVA with Tukey’s multiple comparisons test (*p* < 0.05). Exact *p*-values indicating significant difference between extracts at each concentration are provided in Supplementary Figure S9.

The reducing power assay, which reflects the electron-donating capacity of antioxidants, further confirmed the strong antioxidant potential of *G. lucidum* extracts (Figure 9). At 100 µg/mL, GEE demonstrated the highest reducing power (0.353 ± 0.003), followed by GME (0.176 ± 0.002), GWE (0.164 ± 0.005), and GAE (0.158 ± 0.001). Standard ascorbic acid showed a reducing power of 0.493 ± 0.001 at the same concentration. GEE had significantly higher reducing power than all other extracts at all concentrations (*p* < 0.0001). GWE and GME were significantly different (*p* = 0.0018 at 100 µg/mL), while GWE and GAE were not significantly different at higher dose (*p* > 0.1). Regression analysis showed strong linearity across all samples, with R^2^ values ranging from 0.981 to 0.995, indicating highly consistent dose–response behavior.

### 3.5. MTT-Based Viability Assay in HeLa Cells

**Figure 10:**
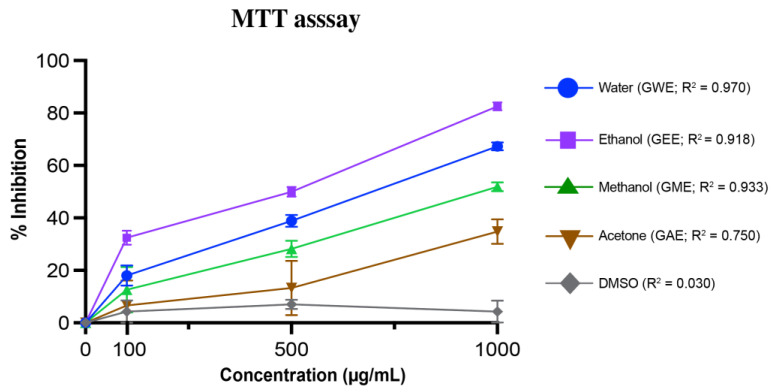
Cytotoxic activity of *G. lucidum* extracts against HeLa cervical cancer cell lines at varying concentration (100, 500, and 1000 µg/mL) with DMSO control. Dose–response regression lines with corresponding R^2^ values are shown. Data are mean ± SD from three biological replicates. Statistical analysis was conducted using one-way ANOVA with Tukey’s multiple comparisons test (*p* < 0.05). Exact *p*-values indicating significant difference between extracts at each concentration are provided in Supplementary Figure S11.

Following the characterization of bioactive compounds and antioxidant activities, the cytotoxic potential of *G. lucidum* extracts was evaluated against human cervical cancer (HeLa) cells via MTT assay (Figure 10). This colorimetric assay measures cellular metabolic activity based on the reduction of MTT reagent to insoluble formazan crystals by mitochondrial dehydrogenases in viable cells (see Supplementary Figure S10). Extracts were tested at concentrations of 100, 500, and 1000 µg/mL, and dose-dependent inhibition of cell viability was observed (Figure 10). At the highest concentration tested (1000 µg/mL), GEE and GWE extracts demonstrated significantly higher cytotoxicity (*p* < 0.0001), suppressing cell proliferation by >65%. Specifically, GEE achieved 83 ± 1% inhibition and GWE 67 ± 1% inhibition. In comparison, GME and GAE showed more moderate inhibition at 1000 µg/mL, with 52 ± 2% and 35 ± 5% inhibition, respectively. DMSO, used as a vehicle control, showed minimal cytotoxicity. The regression analysis showed high R^2^ values for GWE (0.970), GME (0.933), and GEE (0.918), indicating a strong dose–response relationship, whereas GAE showed a weak correlation (R^2^ = 0.750).

### 3.6. IC_50_ Comparison of Extraction Solvents for Antioxidant and Cytotoxicity Activities

**Figure 11:**
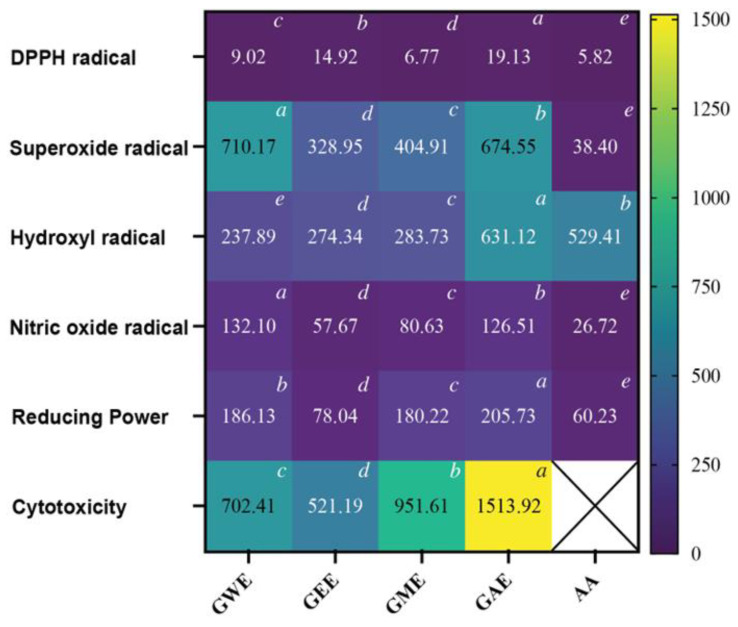
Heatmap of IC_50_ values obtained from various antioxidant and cytotoxic assays. Values are expressed in µg/mL. Within each assay, values with the same letter (a–e) are not significantly different; however, different letters indicate significant differences between solvents (*p* < 0.05). Ascorbic acid: AA.

**Figure 12:**
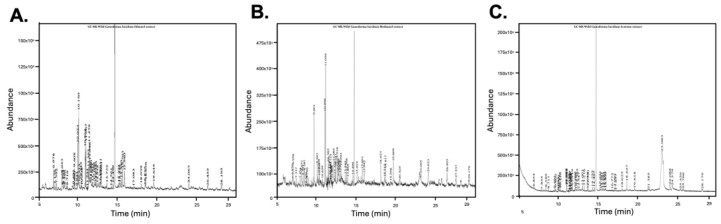
GC-MS chromatograms of *G. lucidum* extracts obtained using different solvents, GEE (**A**), GME (**B**), and GAE (**C**), showing the presence of distinct compounds at various retention times.

The extraction efficiency and bioactive potential of *G. lucidum* varied significantly depending on the solvent used (Figure 11). GEE showed the highest total phenolic (377 ± 9.32 PG/g) and flavonoid (30 ± 1 QE/g) content, while GME showed higher carotenoids (lycopene: 0.067 ± 0.001 mg/g; β-carotene: 0.454 ± 0.000 mg/g) (Figure 11). Antioxidant assays revealed solvent-specific efficacy. All extracts (GWE, GEE, GME, GAE) demonstrated strong DPPH radical scavenging ability, with IC_50_ values ranging between 5.82 and 19.13 µg/mL, comparable to that of ascorbic acid. Notably, GEE was the most potent in superoxide radical scavenging (IC_50_: 328.95 µg/mL), nitric oxide radical scavenging (IC_50_: 57.67 µg/mL), and reducing power (IC_50_: 78.04 µg/mL) assays (Figure 12). Hydroxyl radical inhibition was strongest in GWE (IC_50_: 237.89 µg/mL), closely followed by GEE (274.34 µg/mL).

Cytotoxicity analysis using MTT assay further supported solvent-specific trends. GEE and GWE showed dose-dependent inhibition of HeLa cell viability, with IC_50_ values of 520.19 µg/mL and 702.41 µg/mL, exhibiting moderate activity (Figure 11). GME also showed notable cytotoxicity (951.61 µg/mL), while GAE had the highest IC_50_ (1513.92 µg/mL), indicating lower potency.

Collectively, ethanol was found to be the most effective extraction solvent, capable of extracting phenolic- and flavonoid-rich fractions with broad-spectrum antioxidant and cytotoxic activities, likely due to ethanol extracting a diverse range of polar bioactive compounds [37,38,39]. These findings underscore the strong correlation between ethanol’s high phenolic/flavonoid content and its multi-target bioactivity, positioning it as the optimal solvent for extracting compounds with therapeutic potential against oxidative stress and cancer.

### 3.7. Solvent-Dependent Variation in Bioactive Compounds via GC-MS Profiling

**Figure 13:**
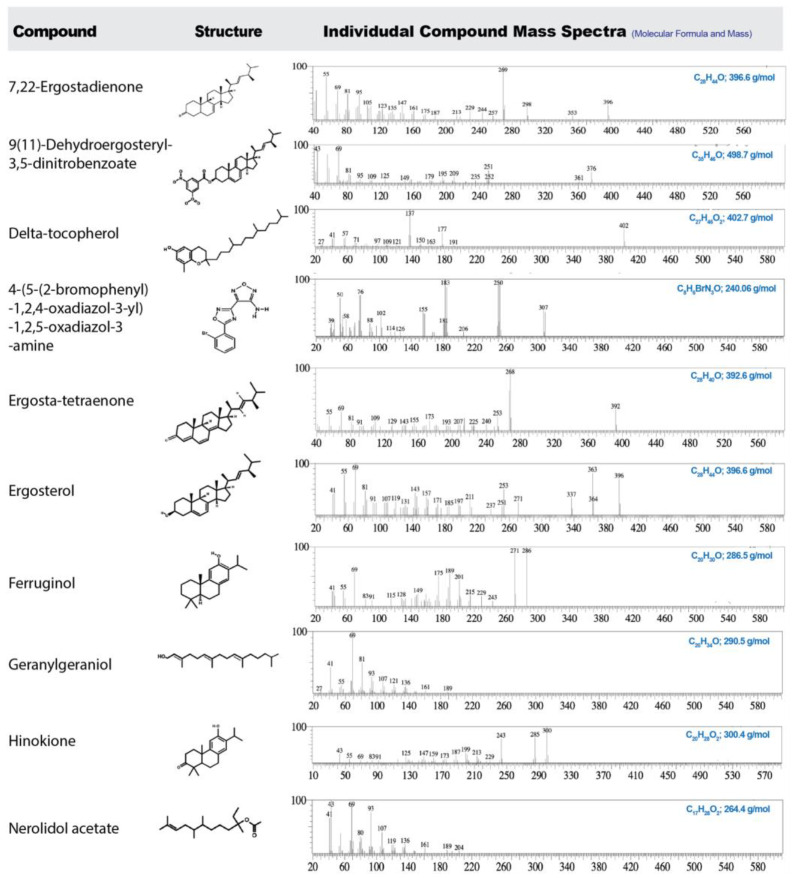
Mass spectra of selected bioactive compounds identified in *G. lucidum* (GEE, GME, and GAE) extracts, along with their chemical structures, molecular formulas, and molecular weights.

GC-MS analysis revealed distinct solvent-dependent chemical profiles in *G. lucidum* extracts. The GC-MS chromatograms of GEE, GME, and GAE are shown in Figure 12, and individual mass spectra with their chemical structure of key pharmacologically active compounds are presented in Figure 13. Detailed information on the identified compounds detected by GC-MS is provided in the Supplementary Materials (Tables S1–S3). The extracts demonstrated unique distributions of sterols, triterpenoids, terpenoids, fatty acids, and polyphenols, highlighting the influence of solvent polarity on bioactive compound composition (Table 3).

Fatty acids dominated ethanol (53.18%) and methanol (48.57%) extracts, with 9,12-octadecadienoic acid (linoleic derivative: 20.60% in ethanol, 14.05% in methanol) and pentadecanoic acid (14.52% in ethanol) as major constituents. Acetone exhibited the lowest fatty acid content (5.64%) but uniquely contained ergosterol (Vitamin D2 precursor) and retinoic acid. Polar protic solvents (ethanol, methanol) efficiently extracted free fatty acids and esters, including (E)-9-octadecenoic acid ethyl ester (oleic derivative: 3.86% in ethanol), while acetone’s mid-polarity favored sterols (7,22-ergostadienone, 9(11)-dehydroergosteryl benzoate) and triterpenoids (ergosta-4,6,8(14),22-tetraen-3-one). Similarly, Hinokione, an abietene diterpene, was detected across solvent extracts (0.9% in GAE, 2.9% in GEE, and 5.5% in GME) (Table 3).

Pharmacologically significant compounds included ferruginol (exclusive to ethanol), nerolidol acetate (methanol-specific), geranylgeraniol (anti-inflammatory terpenoid), and Hinokione (anti-inflammatory and anticancer) (Table 3). Ethanol and methanol extracts had the highest polyphenol, diterpenoid, and fatty acid content, whereas acetone had higher sterols and triterpenoids, demonstrating solvent polarity as a critical determinant of bioactive compound selectivity. These findings show *G. lucidum’s* diverse phytochemical composition and the impact of solvent choice in optimizing targeted metabolite extraction.

## 4. Discussion

This study demonstrated the solvent-dependent extraction of bioactive compounds from *G. lucidum* collected from high-altitude regions of Nepal, supporting our initial hypothesis that unique environmental factors at these altitudes influence the mushrooms’ secondary metabolite profile. The observed variability in extraction yields across different solvents, with acetone yielding the highest, followed by ethanol, methanol, and water, demonstrated the role of solvent polarity in determining extraction efficiency. This observation aligns with established principles of phytochemistry, where solvent polarity dictates the solubilization and subsequent extraction of specific compound classes [33,79,80,81,82,83,84,85].

GEE displayed the highest TPC and TFC, correlating strongly with its superior antioxidant and cytotoxic performance (Figure 4, Figure 5, Figure 6, Figure 7, Figure 8, Figure 9, Figure 10 and Figure 11). GEE’s TPC exceeded that of 62 wild mushrooms previously reported in Nepal and outperformed other *Ganoderma* species and various commercial mushrooms [16,17,86]. GEE also contained approximately 4.7 times more flavonoids than GME, with flavonoid levels surpassing those reported in *G. applanatum* and *G. resinaceum* [86,87]. This suggests that flavonoids likely constitute a significant portion of the total phenolic content in the tested extracts. Collectively, these findings support the efficacy of ethanol in extracting phenolic- and flavonoid-rich fractions with potential nutraceutical value.

Carotenoid extraction with methanol proved to be most effective among the tested solvents due to its polar nature, disrupting the cellular matrix to release hydrophobic carotenoids, consistent with previous reports of carotenoid content in *G. lucidum* [79]. Nevertheless, overall carotenoid yields were relatively low compared to the phenolic and flavonoid content (Figure 3 and Figure 4). The values obtained for GME were higher than those previously reported in Turkish mushrooms [88] and some Indian strains of *G. lucidum* but still lower than those observed in wild Portuguese mushrooms [89]. This reinforces the role of solvent polarity in selective compound recovery. Nonetheless, both β-carotene and lycopene contents were comparatively low when contrasted with the phenolic and flavonoid contents of the same extracts.

Multiple in vitro antioxidant assays, including DPPH, superoxide, hydroxyl, nitric oxide radical scavenging, and reducing power assays, confirmed the strong antioxidant potential of the extracts. All extracts scavenged over 80% of DPPH radicals at 80–100 µg/mL, with GEE and GME showing the strongest activity, likely due to their higher phenolic content. These results indicate better scavenging activity compared to some previously reported wild mushrooms from Nepal, including *G. lucidum* [16,17,86]. Among the assays, superoxide radical scavenging was highest in GEE (72 ± 1%), suggesting strong potential to neutralize ROS via electron donation, primarily attributed to phenolic hydroxyl groups [90]. Similarly, hydroxyl radical scavenging showed potent activity, with IC_50_ values for GWE, GEE, and GME lower than that of ascorbic acid, indicating a potent ability to counter lipid peroxidation and protect against DNA damage, mutagenesis, and oxidative cytotoxicity [91,92,93,94]. GEE also demonstrated strong nitric oxide scavenging (82 ± 1.2%), implying its ability to mitigate nitrosative stress and inhibit peroxynitrite formation, which is implicated in nitrosamine-mediated carcinogenesis within the digestive tract [95]. Reducing power, another key antioxidant indicator, was highest in GEE and significantly exceeded values for *Boletus edulis* and *Pleurotus ostreatus* [89,96,97,98]. This capacity is linked to the hydrogen-donating ability of flavonoids and phenolics [99,100]. Reducing powers of the ethanolic extracts were notably higher than those reported for other *G. lucidum*, *Boletus edulis*, and *Pleurotus ostreatus* [89,96,97,98]. This reducing capacity is likely due to the hydrogen-donating ability of the compounds present in the extracts, which can halt peroxide formation and terminate radical chain reactions [99,100].

The cytotoxicity of the extracts was assessed against HeLa cells using the MTT assay. All extracts demonstrated dose-dependent inhibition of cell viability. At 1000 µg/mL, GEE exhibited the strongest cytotoxic effect (82.53 ± 1.46%), followed by GWE (Figure 11). These results are consistent with earlier reports on the anticancer effects of *G. lucidum*, suggesting that ethanol and water extracts contain compounds that may induce apoptosis, modulate immune responses, and arrest cell cycle progression [79,98,101]. The anti-proliferative effects of *G. lucidum* extracts are well documented in the literature and have been reported in a variety of cancer cell lines, including HeLa (cervical cancer), A549 (lung cancer), LS174 (colon cancer), and MCF-7 (breast cancer) cells [101,102]. As described in recent studies, including the work by Mousavi et al. (2023), these cytotoxic effects are largely attributed to the presence of bioactive compounds such as pentadecanoic acid, 14-methyl ester; hexanoic acid; (Z,Z)-9,12-octadecadienoic acid methyl ester; ergosta-4,6,8(14),22-tetraen-3-one (ergosta-tetraenone); 7,22-ergostadienone; and various *Ganoderma*-derived polysaccharides [101]. Notably, our GC-MS profiling confirmed the presence of these compounds in the solvent extracts of *G. lucidum*, providing mechanistic support for the observed cytotoxicity in HeLa cells and reinforcing their potential therapeutic relevance in cancer treatment.

When IC_50_ values were compared across assays, ethanol emerged as the most effective solvent for extracting multifunctional bioactives (Figure 12). GEE had the lowest IC_50_ values in superoxide (328.95 µg/mL), nitric oxide (57.67 µg/mL), and reducing power (78.04 µg/mL) assays. Although GWE had stronger hydroxyl radical inhibition, GEE consistently performed across multiple assays and demonstrated superior cytotoxicity (IC_50_: 520.19 µg/mL). These findings highlight ethanol’s extraction of polar antioxidant and anticancer agents with broad-spectrum activity. Interestingly, although acetone yielded the highest crude extract mass, it performed poorly in both antioxidant and cytotoxic assays. This apparent discrepancy highlights that a high extraction yield does not necessarily translate to high biological activity. GC-MS analysis revealed that acetone preferentially extracted sterols and retinoids which are structurally large or less bioactive compounds, which, while pharmacologically interesting, may not exert immediate antioxidant or cytotoxic effects at the concentration tested. These findings indicate that extract mass alone is not a reliable indicator of bioactivity.

GC-MS analysis confirmed solvent-specific extraction efficiency, identifying steroids, terpenoids, diterpenoids, triterpenoids, polyphenols, and fatty acids (Table 3 and Supplementary Tables S1–S3). Polyunsaturated fatty acids were most abundant in ethanol and methanol. One of the major bioactive constituents gaining a lot of attention recently and found in all three extracts is hinokione, an abietane-type diterpene known for its significant anticancer and anti-inflammatory activities [70]. Hinokione has been shown to exhibit cytotoxicity against MV-3 and MIAPaCa-2 human cancer cell lines with IC_50_ values of 34.1 and 17.9 µM, respectively, and has demonstrated β-cell regeneration and hypoglycemic effects in zebrafish [70,103,104]. Ferruginol, another abietane diterpenoid with neuroprotective and anticancer activity, was exclusively present in GEE. It has shown antiproliferative activity in melanoma (Sk-MEL28) and various cancer cell lines, including prostate, lung, gastric, and breast cancers, as well as efficacy in CL1-5 xenograft mouse models [59,61,63]. Methanol extract contained nerolidol acetate, a sesquiterpene with antioxidant, antibacterial, anti-biofilm, antifungal, and anticancer properties [73,74,75]. Geranylgeraniol, an anti-inflammatory isoprenoid, was also detected in methanol and ethanol extracts, likely contributing to their antioxidant activity [66,67,68,69]. GAE was rich in ergosterol and retinoic acid, with ergosterol comprising more than two-thirds of the total extracted compounds. As a vitamin D precursor, ergosterol has potential for addressing vitamin D deficiency-associated diseases, including cancers, rheumatoid arthritis, and multiple sclerosis [105]. Estrogenic derivatives such as 7,22-ergostadienone and 9(11)-dehydroergosteryl benzoate, known for their therapeutic applications, were found across all extracts (Table 3). Collectively, the GC-MS dataset underscores the profound impact of solvent choice on the chemical profile of mushroom extracts and the types of bioactive molecules recovered.

The observed solvent-dependent variation in extracted compounds across solvents can be explained by their fundamental physiochemical principles. Ethanol and methanol, both polar protic solvents, can form hydrogen bonds and penetrate cell walls easily, allowing them to extract a broad range of polar bioactives such as phenolics, flavonoids, and fatty acids. Acetone, with its intermediate polarity and aprotic nature, is better at extracting more hydrophobic and structurally rigid compounds. Water, while highly polar, has limited ability to solubilize non-polar or moderately polar compounds. As a result, it primarily extracts hydrophilic constituents, such as polysaccharides, simple phenolics, and certain proteins. These solvent-dependent metabolic signatures not only explain the variation in antioxidant and cytotoxic activities observed across assays but also provide mechanistic insight into the functional contributions of specific compound classes. The selective enrichment of fatty acids, sterols, and terpenoids by distinct solvents offers a strategic basis for tailoring extraction protocols to maximize therapeutic yield.

While our findings provide a strong preliminary basis for the therapeutic potential of *G. lucidum*, several limitations should be addressed in future studies. The cytotoxicity effects were evaluated solely using the MTT assay on a single cancer cell line, without comparison to normal cells. This limits our understanding of extract selectivity and potential off-target effects in normal cells. Follow-up studies should expand cytotoxicity screening to include additional cancer cell lines, such as MCF-7, A549, HT-29, as well as non-cancerous cell lines like HEK293 to assess therapeutic selectivity and safety profiles. Investigating apoptosis-related pathways, cell cycle arrest, or mitochondrial dysfunction will be essential to validate the anticancer properties observed. Building upon these findings, future investigations should also focus on isolating and functionally characterizing the specific bioactive compounds responsible for the observed activities through selective extraction and purification of bioactive candidate compounds. Testing these isolated compounds will provide a clearer understanding of their therapeutic potential.

## 5. Conclusions

Our study aimed to investigate the therapeutic potential of *G. lucidum* from Nepal’s high-altitude regions, and our findings strongly confirm it as a key source of bioactive compounds. Through this work, we have shown that the choice of extraction solvent is critical, significantly impacting not only the yield but also the specific bioactive compounds obtained and, consequently, their biological activities. While acetone yielded the highest amount of crude extract, ethanol and methanol extracts showed higher phenolic and flavonoid content, correlated with high antioxidant activity across a spectrum of in vitro assays. We also showed that high extraction yield does not necessarily translate to high biological activity. The ethanol and water extracts also demonstrated moderate ability to inhibit HeLa cell growth. GC-MS analysis identified a diverse array of beneficial compounds, including fatty acids, sterols like ergosterol, and various terpenoids (diterpenoids, triterpenoids). The specific distribution of these compounds varied depending on the extraction solvents, and they collectively contribute to the observed health benefits. Future research should focus on optimizing extraction methods and characterizing these individual compounds to maximize specific bioactivities, which will be critical for bridging the gap between traditional use and modern applications.

## Figures and Tables

**Table 1:** Summary of the ecological, morphological, anatomical, and taxonomic characteristics of *G. lucidum*.

Parameter	Description
Collection month	September–October
Location	Chandragiri Hill, Kathmandu, Nepal
Elevation	7482 ft (2280 m) above sea level
Coordinates	Latitude: 27.67402° N; Longitude: 85.19874° E
Ecosystem type	Solitary
Substrate	Wood, stump, log, stick, base of tree, bark
Host tree	*Quercus lanata*
Rot type	White-rot
Surrounding trees	Predominantly hardwoods
Basidiocarp size Texture	6–10 cm × 4–16 cm × 1.3 cm Woody to corky
Stipe	Sub-sessile to laterally stipitate, 2–3 cm
Pileus shape	Reniform
Upper surface	Laccate, dark reddish to purplish, yellowish at margins; brittle, soft
Margin	Blunt, rounded, brown-white
Pore surface	Creamy to milky coffee; ~5 pores/mm
Tube layer	2–9 mm long, white turning brown when brushed or aged
Context	9 mm thick, brown, without horny deposition
Cutis type	Thick-walled claviform with diverticula; 35–42 × 6–8.5 µm
Hyphal system	Trimitic: Generative (3.3 µm, hyaline, thin-walled, with clamp); Skeletal (5.8–7.5 µm, brown, thick); Binding (5–7.5 µm, brown)
Basidiospores	8.3–10 × 6.6 µm; yellowish-brown
Identification authority	Prof. Mahesh Kumar Adhikari, Dept. of Plant Resources, Kathmandu

**Table 2:** Percentage yield of various solvent extracts.

Extract	Weight of Sample Before Extraction (gm)	Weight Obtained After Extraction (gm)	% Yield Value
Water	10	0.229	2.29 ^d^
Ethanol	10	0.343	3.43 ^b^
Methanol	10	0.298	2.98 ^c^
Acetone	10	0.501	5.01 ^a^

Values with the same letter (a–d) are not significantly different; different letters indicate significant differences between solvents (*p* < 0.05).

**Table 3:** Summary of key compounds detected by the GC-MS analysis in various solvents with reported pharmacological relevance.

Compound Name	Solvent Extracts (% Area)			Compound Class	Key Pharmacological Relevance	Reference
	GEE	GME	GAE			
7,22-Ergostadienone	3.54	2.90	2.56	Sterol	Antithrombotic activity with cardiovascular benefit; antidiabetic, anticancer, and neuroprotective effects; pro-inflammatory properties (activating Toll-like receptors, cytokines, and chemokines)	[37,40,41,42,43]
9(11)-Dehydroergosteryl 3,5-dinitrobenzoate	2.90	3.13	2.70	Sterol conjugate	Anti-inflammatory; antibacterial (MRSA and *S. aureus*); and cytotoxic properties	[44,45]
δ-Tocopherol	2.13	3.91	0.75	Tocopherol	Antioxidant; anti-inflammatory (primarily Via inhibiting protein kinase C and reducing eicosanoid production); anticancer (both in vitro and in vivo prostate xenograft models); cardioprotective and neuroprotective	[46,47]
4-[5-(2-bromophenyl)-1,2,4-oxadiazol-3-yl]-1,2,5-oxadiazol-3-amine	-	-	0.35	Synthetic heterocycle	Anticancer (potentially via targeting topoisomerase II relaxation activity); antibacterial; anti-inflammatory; analgesic properties; antioxidant	[48,49,50,51,52]
Ergosta-tetraenone	3.86	-	1.67	Sterol derivative	Anticancer (Via G_2_/M arrest and apoptosis induction); nephroprotection (mitigation of renal damage in mouse model); anti-inflammatory	[53,54,55]
Ergosterol	-	-	73.99	Sterol	Vitamin D2 precursor; lipid soluble antioxidant; anticancer effects (cell cycle arrest and modulates Wnt/β-catenin signaling pathway); antimicrobial; antidiabetic; immunomodulatory effects	[56,57,58]
Ferruginol	3.18	-	-	Abietane diterpene	Anticancer (apoptosis induction in melanoma, prostate, lung, and ovarian cancer cells); neuroprotective (reduces α-synuclein toxicity and restores LTP in Alzheimer’s models); cardioprotective (both in vitro and in vivo models); antimicrobial and antiviral	[59,60,61,62,63,64,65]
Geranylgeraniol	5.26	-	0.89	Diterpenoid alcohol	Anti-inflammatory (NF-κB inhibition; decreased IL-1β, TNF-α, IL-6, COX-2); pain relief; bone and muscle support (muscle regeneration and prevents bisphosphonate-related bone damage); antimicrobial activity; hormonal balance; glucose homeostasis	[66,67,68,69]
Hinokione	2.9	5.5	0.9	Abietane diterpene	Anticancer; anti-inflammatory; hypoglycemic and β-Cell regenerative properties (promotes β-cell differentiation and improved glycemia in zebrafish); antibacterial; antioxidant	[70,71,72]
Nerolidol acetate	-	1.70	-	Sesquiterpene ester	Anticancer; anti-inflammatory; neuroprotective; antimicrobial; antifungal; antioxidant	[73,74,75]
Retinoic acid	-	-	0.50	Retinoid	Acne and photoaging (promotes cell differentiation and skin repair); anticancer (induces differentiation of malignant promyelocytes in acute promyeloid leukemia); neuroprotective	[76,77,78]

## Data Availability

Data are contained within the article and Supplementary Materials.

## Supplementary Materials

The following supporting information can be downloaded at: https://www.mdpi.com/article/10.3390/cimb47080624/s1.

## Abbreviations

The following abbreviations are used in this manuscript:

GWE	*Ganoderma lucidum* water extract
GEE	*Ganoderma lucidum* ethanol extract
GME	*Ganoderma lucidum* methanol extract
GAE	*Ganoderma lucidum* acetone extract
TPC	Total phenolic content
TFP	Total flavonoid content
DPPH	2,2-diphenyl-1-picryl-hydrazyl
MTT	3-(4,5-dimethylthiazol-2-yl)-2,5-diphenyl tetrazolium bromide
GC-MS	Gas chromatography–mass spectrometry
LTP	Long-term potentiation
MRSA	Methicillin-resistant *Staphylococcus aureus*
HeLa	Human cervical carcinoma cell lines
