# Supplementary material for: Evaluation of Bioactive Compounds, Antioxidant Activity, and Anticancer Potential of Wild Ganoderma lucidum Extracts from High-Altitude Regions of Nepal
Source: Curr Issues Mol Biol. 2025 Aug 5;47(8):624. doi: 10.3390/cimb47080624 (PMC12384079; doi:10.3390/cimb47080624)
Supplement: Supplementary file 1 [file cimb-47-00624-s001.zip › cimb-3765836-supplementary.pdf]

Evaluation of Bioactive Compounds, Antioxidant Activity, and Anticancer Potential of Wild *Ganoderma lucidum* Extracts from High-Altitude Regions of Nepal

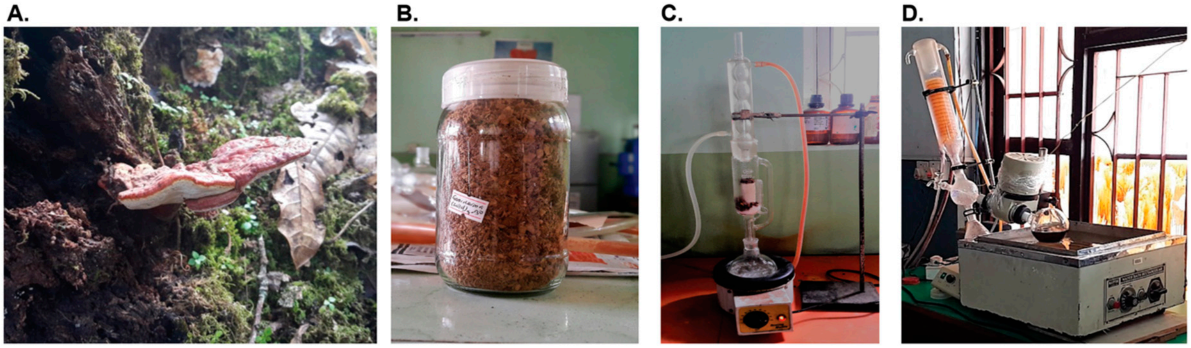

Supplementary Figure S1. Visual representation of key stages in the preparation of *G. ludicum* extracts. *G. lucidum* in its natural habitat (A) and its dried powder (B) Soxhlet apparatus setup (C) and rotary vaccum evaporator employed for solvent removal (D).

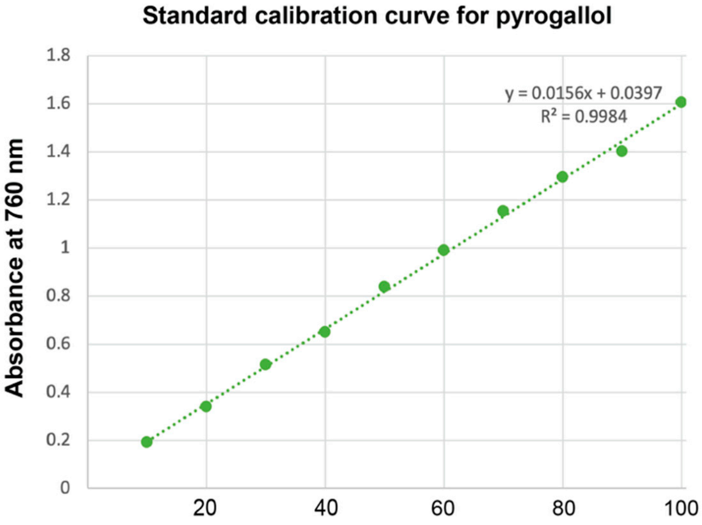

Supplementary Figure S2. Linear regression equation derived from the standard calibration curve of pyrogallol, used to determine the total phenolic content of *G. lucidum* extracts.

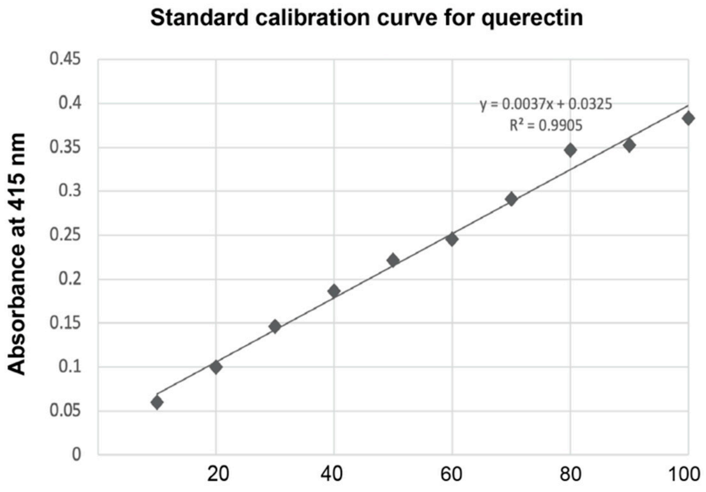

Supplementary Figure S3. Linear regression equation derived from the standard calibration curve of querectin, used to determine the total flavonoid content of *G. lucidum* extracts.

	TPC		TFP		β-carotene		Lycopene	
Comparision	Summary	p-value	Summary	p-value	Summary	p-value	Summary	p-value
GWE vs. GEE	****	<0.0001	****	<0.0001	*	0.0174	***	0.0005
GWE vs. GME	ns	0.9987	****	<0.0001	****	<0.0001	****	<0.0001
GWE vs. GAE	***	0.0005	****	<0.0001	***	0.0007	ns	0.9156
GEE vs. GME	****	<0.0001	****	<0.0001	****	<0.0001	****	<0.0001
GEE vs. GAE	****	<0.0001	****	<0.0001	****	<0.0001	***	0.0002
GME vs. GAE	***	0.0004	**	0.0096	****	<0.0001	****	<0.0001

One-way ANOVA followed by Tukey's multiple comparision test

Supplementary Figure S4. Adjusted p-values for comparision of total phenolic content (TPC), total flavonoid content (TFC), β-carotene, and lycopene content across different *G. lucidum* extracts. Statistical analysis was performed using one-way ANOVA followed by Tukey's multiple comparisons test ($p < 0.05$).

DPPH (2, 2-diphenyl-1-picryl-hydrazyl) assay — **One-way ANOVA followed by Tukey's multiple comparision test**

	20 µg/ml		40 µg/ml		60 µg/ml		80 µg/ml		100 µg/ml	
**Comparision**	**Summary**	**p-value**	**Summary**	**p-value**	**Summary**	**p-value**	**Summary**	**p-value**	**Summary**	**p-value**
GWE vs. GEE	ns	0.1936	ns	0.7894	ns	0.8211	ns	0.2364	ns	0.163
GWE vs. GME	ns	0.7879	*	0.0364	ns	0.139	**	0.0063	**	0.0027
GWE vs. GAE	**	0.0057	ns	0.4882	ns	0.8617	ns	0.1727	ns	0.0555
GWE vs. AA	***	0.0008	****	<0.0001	*	0.0166	**	0.0024	***	0.0007
GEE vs. GME	ns	0.7164	**	0.0066	ns	0.5512	ns	0.1946	ns	0.1205
GEE vs. GAE	ns	0.2129	ns	0.9797	ns	>0.9999	ns	0.9994	ns	0.9496
GEE vs. AA	****	<0.0001	****	<0.0001	ns	0.0856	ns	0.0699	*	0.0271
GME vs. GAE	*	0.0313	**	0.003	ns	0.5017	ns	0.2648	ns	0.3281
GME vs. AA	***	0.0002	**	0.0047	ns	0.6505	ns	0.9558	ns	0.863
GAE vs. AA	****	<0.0001	****	<0.0001	ns	0.0745	ns	0.0981	ns	0.0812

Supplementary Figure S5. Adjusted p-values for comparisions of DPPH radical scavenging activity amoung different *G. lucidum* extracts at varying concentrations. Statistical analysis was performed using one-way ANOVA followed by Tukey's multiple comparisons test ($p < 0.05$). Significant difference between solvents extracts at each concentration are summarized by significance level (asterisks) and exact p-value.

Superoxide radical scavenging assay — **One-way ANOVA followed by Tukey's multiple comparision test**

	100 µg/ml		200 µg/ml		300 µg/ml		400 µg/ml		500 µg/ml	
**Comparision**	**Summary**	**p-value**	**Summary**	**p-value**	**Summary**	**p-value**	**Summary**	**p-value**	**Summary**	**p-value**
GWE vs. GEE	****	<0.0001	****	<0.0001	****	<0.0001	**	0.0023	****	<0.0001
GWE vs. GME	**	0.0016	***	0.0005	****	<0.0001	**	0.0066	****	<0.0001
GWE vs. GAE	**	0.0048	ns	0.1268	ns	0.1882	ns	>0.9999	ns	0.9493
GWE vs. AA	****	<0.0001	****	<0.0001	****	<0.0001	****	<0.0001	****	<0.0001
GEE vs. GME	ns	0.226	*	0.0303	ns	0.9832	ns	0.9403	****	<0.0001
GEE vs. GAE	****	<0.0001	****	<0.0001	****	<0.0001	**	0.0021	****	<0.0001
GEE vs. AA	****	<0.0001	****	<0.0001	****	<0.0001	****	<0.0001	****	<0.0001
GME vs. GAE	****	<0.0001	****	<0.0001	****	<0.0001	**	0.0061	****	<0.0001
GME vs. AA	****	<0.0001	****	<0.0001	****	<0.0001	****	<0.0001	****	<0.0001
GAE vs. AA	****	<0.0001	****	<0.0001	****	<0.0001	****	<0.0001	****	<0.0001

Supplementary Figure S6. Adjusted p-values for comparisions of superoxide radical scavenging activity amoung different *G. lucidum* extracts at varying concentrations. Statistical analysis was performed using one-way ANOVA followed by Tukey's multiple comparisons test ($p < 0.05$). Significant difference between solvents extracts at each concentration are summarized by significance level (asterisks) and exact p-value.

Hydroxyl radical scavenging assay — **One-way ANOVA followed by Tukey's multiple comparision test**

	100 µg/ml		200 µg/ml		300 µg/ml		400 µg/ml		500 µg/ml	
**Comparision**	**Summary**	**p-value**	**Summary**	**p-value**	**Summary**	**p-value**	**Summary**	**p-value**	**Summary**	**p-value**
GWE vs. GEE	****	<0.0001	****	<0.0001	****	<0.0001	****	<0.0001	****	<0.0001
GWE vs. GME	****	<0.0001	****	<0.0001	****	<0.0001	****	<0.0001	****	<0.0001
GWE vs. GAE	****	<0.0001	****	<0.0001	****	<0.0001	****	<0.0001	****	<0.0001
GWE vs. AA	****	<0.0001	****	<0.0001	****	<0.0001	****	<0.0001	****	<0.0001
GEE vs. GME	****	<0.0001	ns	0.5803	ns	0.4435	****	<0.0001	ns	0.2855
GEE vs. GAE	****	<0.0001	****	<0.0001	****	<0.0001	****	<0.0001	****	<0.0001
GEE vs. AA	****	<0.0001	****	<0.0001	****	<0.0001	****	<0.0001	****	<0.0001
GME vs. GAE	****	<0.0001	****	<0.0001	****	<0.0001	****	<0.0001	****	<0.0001
GME vs. AA	****	<0.0001	****	<0.0001	****	<0.0001	****	<0.0001	****	<0.0001
GAE vs. AA	ns	0.0511	****	<0.0001	****	<0.0001	****	<0.0001	****	<0.0001

Supplementary Figure S7. Adjusted p-values for comparisions of hydroxyl radical scavenging activity amoung different *G. lucidum* extracts at varying concentrations. Statistical analysis was performed using one-way ANOVA followed by Tukey's multiple comparisons test ($p < 0.05$). Significant difference between solvents extracts at each concentration are summarized by significance level (asterisks) and exact p-value.

Nitric Oxide radical scavenging assay **One-way ANOVA followedd by Tukey's multiple comparision test**

	20 μg/ml		40 μg/ml		60 μg/ml		80 μg/ml		100 μg/ml	
**Comparision**	**Summary**	**p-value**	**Summary**	**p-value**	**Summary**	**p-value**	**Summary**	**p-value**	**Summary**	**p-value**
GWE vs. GEE	***	0.0001	****	<0.0001	****	<0.0001	****	<0.0001	****	<0.0001
GWE vs. GME	ns	0.9574	**	0.0036	*	0.0368	****	<0.0001	***	0.0003
GWE vs. GAE	ns	0.9644	ns	>0.9999	ns	0.9984	ns	0.4281	ns	>0.9999
GWE vs. AA	****	<0.0001	****	<0.0001	****	<0.0001	****	<0.0001	****	<0.0001
GEE vs. GME	***	0.0002	ns	0.0813	**	0.0025	***	0.0004	**	0.002
GEE vs. GAE	***	0.0002	****	<0.0001	****	<0.0001	****	<0.0001	****	<0.0001
GEE vs. AA	****	<0.0001	****	<0.0001	****	<0.0001	****	<0.0001	ns	0.0523
GME vs. GAE	ns	>0.9999	**	0.0035	*	0.0238	***	0.0005	***	0.0004
GME vs. AA	****	<0.0001	****	<0.0001	****	<0.0001	****	<0.0001	****	<0.0001
GAE vs. AA	****	<0.0001	****	<0.0001	****	<0.0001	****	<0.0001	****	<0.0001

Supplementary Figure S8. Adjusted p-values for comparisions of nitric oxide radical scavenging activity amoung different *G. lucidum* extracts at varying concentrations. Statistical analysis was performed using one-way ANOVA followed by Tukey's multiple comparisons test ($p < 0.05$). Significant difference between solvents extracts at each concentration are summarized by significance level (asterisks) and exact p-value.

Reducing power assay **One-way ANOVA followed by Tukey's multiple comparision test**

	20 μg/ml		40 μg/ml		60 μg/ml		80 μg/ml		100 μg/ml	
**Comparision**	**Summary**	**p-value**	**Summary**	**p-value**	**Summary**	**p-value**	**Summary**	**p-value**	**Summary**	**p-value**
GWE vs. GEE	****	<0.0001	****	<0.0001	****	<0.0001	****	<0.0001	****	<0.0001
GWE vs. GME	**	0.0015	**	0.0031	*	0.0103	****	<0.0001	**	0.0018
GWE vs. GAE	**	0.0079	*	0.0227	ns	0.6814	ns	>0.9999	ns	0.1101
GWE vs. AA	****	<0.0001	****	<0.0001	****	<0.0001	****	<0.0001	****	<0.0001
GEE vs. GME	****	<0.0001	****	<0.0001	****	<0.0001	****	<0.0001	****	<0.0001
GEE vs. GAE	****	<0.0001	****	<0.0001	****	<0.0001	****	<0.0001	****	<0.0001
GEE vs. AA	***	0.0001	*	0.032	****	<0.0001	****	<0.0001	****	<0.0001
GME vs. GAE	ns	0.7607	ns	0.674	ns	0.0798	****	<0.0001	****	<0.0001
GME vs. AA	**	0.0028	****	<0.0001	****	<0.0001	****	<0.0001	****	<0.0001
GAE vs. AA	***	0.0006	****	<0.0001	****	<0.0001	****	<0.0001	****	<0.0001

Supplementary Figure S9. Adjusted p-values for comparisions of reducing power activity amoung different *G. lucidum* extracts at varying concentrations. Statistical analysis was performed using one-way ANOVA followed by Tukey's multiple comparisons test ($p < 0.05$). Significant difference between solvents extracts at each concentration are summarized by significance level (asterisks) and exact p-value.

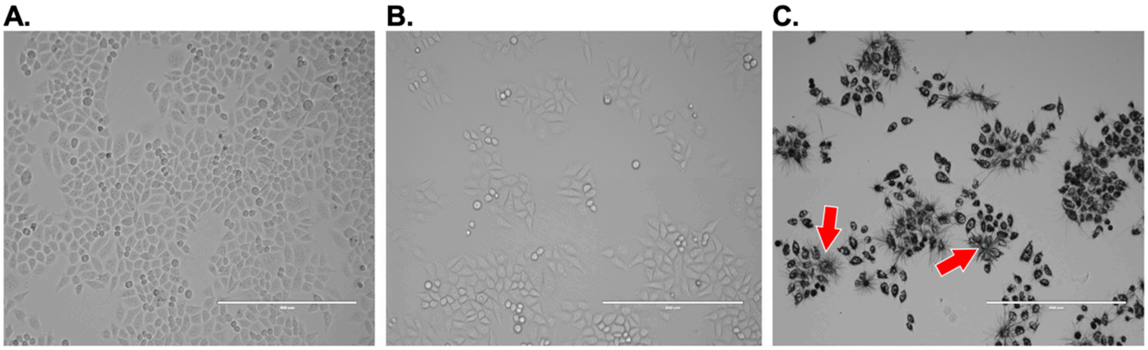

Supplementary Figure S10. Bright-field microscopy images of HeLa cells. Untreated HeLa cell with normal morphology (**A**), HeLa cell treated with *G. lucidum* extract showing reduced proliferation 24 h post treatment (**B**), and formation of intracellular formazan crystal (indicated by red arrow), reflecting the metabolic activity in treated cells (**C**).

MTT assay — One-way ANOVA followed by Tukey's multiple comparision test

	100 µg/ml		500 µg/ml		1000 µg/ml	
**Comparision**	**Summary**	**p-value**	**Summary**	**p-value**	**Summary**	**p-value**
DMSO vs. GWE	ns	0.1395	***	0.0001	****	<0.0001
DMSO vs. GEW	**	0.0022	****	<0.0001	****	<0.0001
DMSO vs. GME	ns	0.5362	**	0.0033	****	<0.0001
DMSO vs. GAE	ns	0.9909	ns	0.5819	***	0.0001
GWE vs. GEW	ns	0.1142	ns	0.127	*	0.0191
GWE vs. GME	ns	0.8348	ns	0.1469	*	0.0179
GWE vs. GAE	ns	0.2622	***	0.0008	****	<0.0001
GEW vs. GME	*	0.0232	**	0.0026	***	0.0001
GEW vs. GAE	**	0.0042	****	<0.0001	****	<0.0001
GME vs. GAE	ns	0.7805	*	0.0308	**	0.0093

Supplementary Figure S11. Adjusted p-values for comparisions amoung different *G. lucidum* extracts and the DMSO control at each concentration. Statistical analysis was performed using one-way ANOVA followed by Tukey's multiple comparisons test ($p < 0.05$). Significant difference between solvents extracts at each concentration are summarized by significnace level (asterisks) and exact p-value.

Supplementary Table S1. List of compounds in ethanol extract of wild *G. lucidum* detected by GC-MS analysis

Compound Name	MF	MW (g/mol)	Classification	RT (min)	RI	Area %	Base m/z
(E)-9-octadecenoic-acid ethyl ester	$C_{20}H_{38}O_2$	310.52	Fatty-acid ester	11.392	2185	3.86	55.05
(Z)-β-Farnesene	$C_{15}H_{24}$	204.35	Terpenoid (sesquiterpene)	15.703	1350	6.08	69.1
Methyl 11,14-eicosadienoic acid	$C_{21}H_{38}O_2$	322.53	Fatty-acid ester	11.546	2292	5.31	67.1
3,4,4a,9,10,10a-Hexahydro-2-phenanthrenone	$C_{14}H_{16}O$	200.28	Polycyclic ketone	19.645	2396	2.90	300.2
2-(7-hydroxymethyl-3,11-dimethyl-dodeca-2,6,10-trien-1-yl)ethan-1-ol	$C_{17}H_{30}O_2$	266.42	Terpenoid (diterpene)	15.338	2396	3.22	160.15
Dimethyl chromanol	$C_{11}H_{14}O_2$	178.23	Phenolic benzopyran	18.039	2923	2.13	402.45
Phytadiene isomer	$C_{20}H_{40}$	280.53	Terpenoid hydrocarbon	11.903	1954	1.50	70.05
Methylheptenone	$C_8H_{14}O$	126.2	Aliphatic ketone	7.342	2704	1.54	43.05
5,10-Diethoxy-2,3,7,8-tetrahydro-1H,6H-dipyrrolo[1,2-a:1′,2′-d]pyrazine	$C_{14}H_{22}N2O_2$	252.31	Hetero-aromatic	12.275	1898	0.82	70.1
7,22-ergostadienone	$C_{28}H_{44}O$	396.65	Steroid / ketone	24.063	2623	3.54	269.25
7-Oxocholesteryl isocaproate	$C_{33}H_{54}O_3$	498.78	Sterol ester	26.455	3241	2.27	43.05
9(11)-Dehydroergosteryl benzoate	$C_{35}H46O_2$	504.78	Sterol ester	18.636	3374	2.90	251.25
2-Hydroxy-linoleic acid	$C_{18}H_{32}O_3$	296.45	Oxidised fatty acid	11.479	2713	20.60	67.1
6,6-Dimethyl-2-methylene-7-(3-oxobutyl)hept-3-enoic acid	$C_{16}H24O4$	210.27	Terpenoid acid	12.098	1976	1.91	43.05
Ergosta-4,6,8(14),22-tetraen-3-one	$C_{28}H_{40}O$	394.64	Steroid / ketone	28.195	2644	3.86	268.25
Ferruginol	$C_{20}H_{30}O$	286.45	Phenolic diterpenoid	17.083	2225	3.18	229.2
Geranylgeraniol	$C_{20}H_{34}O$	290.48	Terpenoid (diterpene)	15.94	2192	5.26	69.1
Hexadecanal	$C_{16}H_{32}O$	240.42	Long-chain aldehyde	8.234	1800	1.04	43.1
Methyl 2-oxopalmitate	$C_{17}H_{34}O_3$	284.44	Oxo-fatty-acid ester	10.443	1878	8.89	57.1
Methyl pyroglutamate	$C_6H_9NO_3$	143.14	Cyclic amino-acid ester	7.136	1091	0.95	84.1
Oleamide diethanolamide	$C_{22}H_{43}N_2O_3$	356.54	Fatty-acid amide	15.54	2902	2.54	88.2
Pentadecanoic acid	$C_{17}H_{34}O_2$	242.4	Fatty acid	10.023	1814	14.52	43.1
Hexahydro-pyrrolo[1,2-a]pyrazine-1,4-dione	$C11H_{18}N_2O_2$	142.16	Diketopiperazine	11.68	1699	1.18	70.05

MF: Molecular Formula, MW: Molecular Weight (g/mol), RT: Retention Time in minutes, m/z: mass-to-charge ratio, RI: Retention Index

Supplementary Table S2. List of compounds in methanol extract of wild *G. lucidum* detected by GC-MS analysis

Compound Name	MF	MW (g/mol)	Classification	RT (min)	RI	Area %	Base m/z
(E)-9-octadecenoic-acid ethyl ester	$C_{20}H_{38}O_2$	310.52	Fatty-acid ester	11.385	2185	1.54	55.1
Methyl 10-octadecenoate	$C_{19}H_{36}O_2$	296.49	Fatty-acid ester	10.958	2085	12.85	55.1
13-Docosenoic acid, methyl ester	$C_{23}H_{44}O_2$	352.6	Fatty-acid ester	12.876	2483	1.18	43.05
8-Methyl-1-decene	$C_{11}H_{22}$	154.29	Alkene (hydrocarbon)	11.868	1041	1.03	70.1
Pentadecyl 2-bromopropanoate	$C_{18}H_{35}BrO_2$	348.45	Halo-fatty-acid ester	8.221	2210	1.91	43.1
3,4,4a,9,10,10a-Hexahydro-2-phenanthrenone	$C_{14}H_{16}O$	200.28	Polycyclic ketone / aromatic	19.609	2396	5.54	300.3
δ-Tocopherol	$C_{13}H_{16}O_2$	204.26	Benzopyran / phenolic	18.023	2923	3.91	137.2
3,4-Dihydroxyacetophenone	$C_8H_8O_3$	152.15	Phenolic ketone	8.031	1470	3.32	137.15
4-Methyl-3-hepten-2-one	$C_8H_{14}O$	126.2	Unsat. ketone / volatile	7.337	938	1.40	43.05
4-Hydroxy-β-ionone	$C_{13}H_{20}O_2$	208.3	Nor-isoprenoid	12.646	1646	2.25	43.05
6-Hydroxy-7-isopropyl-1,4a-dimethyl-1,2,3,4-tetrahydronaphthalene	$C_{20}H_{28}O_2$	300	Terpenoid alcohol	20.425	2396	1.18	201.15
7,22-Ergostadienone	$C_{28}H_{44}O$	396.65	Steroid/sterone	24.023	2623	2.90	269.25
9(11)-Dehydroergosteryl benzoate	$C_{35}H_{46}O_2$	498.78	Sterol ester	18.617	3374	3.13	251.25
Methyl linoleate	$C_{19}H_{34}O_2$	294.47	Poly-unsat. fatty-acid ester	11.056	2093	14.05	67.1
9,11-Epoxy-androstan-3,17-dione	$C_{19}H_{26}O_3$	302.43	Steroid ketone	13.864	2135	1.11	41.1
2-Hydroxy-bicyclo[3.1.0]hexan-6-yl methanol	$C_7H_{10}O_2$	126.17	Bicyclic alcohol	12.727	920	2.29	43.05
Methyl behenate	$C_{23}H_{46}O_2$	354.61	Saturated fatty-acid ester	13.542	2475	1.44	74.05
Ergosta-5,7-dien-3β-ol	$C_{28}H_{46}O$	398.66	Sterol (unsat.)	23.095	2642	1.48	43.1
Ergosta-7,22-dien-3β-ol acetate	$C30H_{48}O_2$	440	Sterol ester	26.403	2779	2.30	43.05
Ethyl 1-hexyl-4-hydroxy-2-oxo-1H-quinoline-3-carboxylate	$C_{18}H_{23}NO_4$	317.39	Hetero-aromatic	19.298	2552	1.63	187.15
3,7,11,15-Tetramethyl-hexadeca-2,6,10,14-tetraen-1-ol	$C_{20}H_{34}O$	290.48	Diterpene alcohol	15.691	2192	2.75	69.1
Methyl palmitate	$C_{17}H_{34}O_2$	270.45	Saturated fatty-acid ester	9.601	1878	8.65	74.05
N-(2-Methyl-2H-tetrazol-5-yl)-acetamide	$C_4H_7N_5O$	141.12	Heterocycle amide	6.956	0	2.94	43.05
Nerolidyl acetate	$C_{17}H_{28}O_2$	264	Terpenoid	11.769	1754	1.70	93.1
Pentadecanoic acid	$C_{15}H_{30}O_2$	242.4	Saturated fatty acid	9.991	1869	4.70	43.1
13β-Methyl-13-vinyl-podocarp-7-en-3-one	$C_{20}H_{30}O$	286	Diterpenoid ketone	18.512	2097	1.42	43.1
Pregnan-3α,17α-diol-20-one	$C_{21}H_{34}O_3$	334.49	Steroid	27.537	2404	2.04	43.1
Stigmastane	$C_{29}H_{52}$	400.68	Steroid hydrocarbon	29.179	2532	1.69	43.05
Methyl lignocerate	$C_{25}H_{50}O_2$	382.66	Saturated fatty-acid ester	15.019	2674	1.11	74.05
Methyl 12-methyltetradecanoate	$C_{16}H_{32}O_2$	256.42	Branched fatty-acid ester	12.26	1715	3.05	70.1
1′-Acetoxy-3-methyl-3-demethylene-xanthatin	$C_{17}H_{24}O_5$	262.3	Sesquiterpene lactone	12.085	2257	3.50	43.05

MF: Molecular Formula, MW: Molecular Weight (g/mol), RT: Retention Time in minutes, m/z: mass-to-charge ratio, RI: Retention Index

Supplementary Table S3. List of compounds in acetone extract of wild *G. lucidum* detected by GC-MS analysis

Compound Name	MF	MW (g/mol)	Classification	RT (min)	RI	Area %	Base m/z
(E)-9-octadecenoic-acid ethyl ester	$C_{20}H_{38}O_2$	310.52	Fatty-acid ester	11.38	2185	0.99	55.1
ergosta-5,7,22-trien-3-ol	$C_{28}H_{44}O$	396.65	Sterol	24.379	2650	2.73	43.1
4-[5-(2-Bromophenyl)-1,2,4-oxadiazol-3-yl]-1,2,5-oxadiazol-3-amine	$C_{10}H_6BrN_5O$	284.06	Hetero-aromatic (N/O)	11.327	2432	0.35	149.2
Methyl cis-11-octadecenoate	$C_{19}H_{36}O_2$	296.49	Fatty-acid ester	12.547	2085	0.60	43.05
2-(7-Hydroxymethyl-3,11-dimethyl-dodeca-2,6,10-trien-1-yl)ethan-1-ol	$C_{17}H_{30}O_2$	266.42	Diterpene alcohol	15.363	2706	0.78	147.2
δ-Tocopherol	$C_{27}H_{46}O_2$	178.23	Benzopyran phenolic	18.025	2923	0.75	137.15
4-(1-Hydroperoxy-2,2-dimethyl-6-methylene-cyclohexyl)but-2-en-1-ol	$C_{14}H_{22}O_3$	230.31	Oxidised terpenoid	12.092	1835	0.71	43.05
2,6-Di-tert-butyl-4-azido-2-nitrobutyric acid	$C_{19}H_{28}N_4O_5$	287.31	Alkyl-nitro compound	11.563	0	0.39	43.05
7,22-ergostadienone	$C_{28}H_{44}O$	396.65	Steroid/ketone	24.088	2623	2.56	269.25
7-Cholesten-3-one	$C_{27}H_{44}O$	384.64	Steroid ketone	25.429	2580	1.35	43.05
9(11)-Dehydroergosteryl benzoate	$C_{35}H_{46}O_2$	504.78	Sterol ester	18.627	3374	2.70	251.25
Methyl linoleate	$C_{19}H_{34}O_2$	294.47	Fatty-acid ester	11.055	2093	1.81	67.1
Androst-7-ene-2,3,14,17-tetrol-6,17-dione	$C_{19}H_{26}O_5$	318.41	Steroid polyol/ketone	25.766	2649	1.53	43.05
1-Pentyl-2-propyl-cyclopentane	$C_{13}H_{26}$	180.33	Hydrocarbon	9.933	1318	0.50	55.1
Ergosta-4,6,8(14),22-tetraen-3-one	$C_{28}H_{40}O$	394.64	Steroid/ketone	28.176	2644	1.67	268.25
Ergosterol	$C_{28}H_{44}O$	396.65	Sterol	23.083	2650	73.99	69.1
3,7,11-trimethyl-2,6,10-dodecatrien-1-ol	$C_{15}H_{26}O$	222.37	Terpenoid	15.85	1634	0.53	69.1
Geranylgeraniol	$C_{20}H_{34}O$	290.48	Terpenoid	15.693	2192	0.89	69.1
N-(4-Ethyl-5-methyl-heptanoyl)amide	$C_{10}H_{21}NO$	241.37	Fatty-amide	12.164	1296	0.50	59.05
Methyl palmitate	$C_{17}H_{34}O_2$	270.45	Fatty-acid ester	9.602	1878	0.46	74.1
n-Hexadecanoic acid	$C_{16}H_{32}O_2$	256.42	Fatty acid	9.991	1968	1.38	43.05
Pentadecanal	$C_{21}H_{32}O$	226.39	Long-chain aldehyde	13.649	1701	0.46	43.05
Octahydro-phenanthrene	$C_{14}H_{22}$	190.32	Polycyclic hydrocarbon	19.618	2193	0.89	300.25
All-trans-Retinoic acid	$C_{20}H_{28}O_2$	302.45	Acyclic carotenoid / retinoid	21.385	2352	0.50	309.3
7-Hexyl-tridecane	$C_{19}H_{40}$	268.52	Hydrocarbon	17.097	1846	0.60	57.1
Diethyl tridecanedioate	$C_{17}H_{32}O_4$	298.42	Dicarboxylic-acid ester	11.433	2046	0.39	163.15

MF: Molecular Formula, MW: Molecular Weight (g/mol), RT: Retention Time in minutes, m/z: mass-to-charge ratio, RI: Retention Index

GC-MS operating conditions:

For GC analysis
Solvent Cut time: 4 minute
Oven Temperature Program

For MS analysis
Start m/z: 40.00
End m/z: 600.00

Rate	Temperature (°C)	Hold Time (min)
**-**	100	0.00
**15.00**	250	1.00
**30.00**	280	2.00
**15.00**	300	15.00
